# Spatial multi-omics analysis of the microenvironment in traumatic spinal cord injury: a narrative review

**DOI:** 10.3389/fimmu.2024.1432841

**Published:** 2024-08-29

**Authors:** Run Peng, Liang Zhang, Yongqi Xie, Shuang Guo, Xinqi Cao, Mingliang Yang

**Affiliations:** ^1^ School of Rehabilitation Medicine, Capital Medical University, Beijing, China; ^2^ Department of Rehabilitation, Guangdong Provincial People’s Hospital, Guangdong Academy of Medical Sciences, Guangzhou, China; ^3^ Department of Spinal and Neural Functional Reconstruction, China Rehabilitation, Research Center, Beijing, China; ^4^ Center of Neural Injury and Repair, Beijing Institute for Brain Disorders, Beijing, China; ^5^ Beijing Key Laboratory of Neural Injury and Rehabilitation, Beijing, China

**Keywords:** single cell, single cell RNA sequencing, spatial transcriptomics, spatial multi-omics, microenvironment, immune microenvironment, heterogeneity, spinal cord injury

## Abstract

Traumatic spinal cord injury (tSCI) is a severe injury to the central nervous system that is categorized into primary and secondary injuries. Among them, the local microenvironmental imbalance in the spinal cord caused by secondary spinal cord injury includes accumulation of cytokines and chemokines, reduced angiogenesis, dysregulation of cellular energy metabolism, and dysfunction of immune cells at the site of injury, which severely impedes neurological recovery from spinal cord injury (SCI). In recent years, single-cell techniques have revealed the heterogeneity of multiple immune cells at the genomic, transcriptomic, proteomic, and metabolomic levels after tSCI, further deepening our understanding of the mechanisms underlying tSCI. However, spatial information about the tSCI microenvironment, such as cell location and cell-cell interactions, is lost in these approaches. The application of spatial multi-omics technology can solve this problem by combining the data obtained from immunohistochemistry and multiparametric analysis to reveal the changes in the microenvironment at different times of secondary injury after SCI. In this review, we systematically review the progress of spatial multi-omics techniques in the study of the microenvironment after SCI, including changes in the immune microenvironment and discuss potential future therapeutic strategies.

## 1 Introduction

The pathological mechanism of traumatic spinal cord injury (tSCI) can be divided into primary and secondary injuries based on their pathological process (1). The former is injuries caused by external forces acting directly or indirectly on the spinal cord, which can lead to severe destruction of spinal cord structure and function. Currently, there is no effective clinical treatment. Secondary injuries are caused by a variety of factors following the primary spinal cord injury (SCI), including vascular dysfunction, edema, ischemia, excitotoxicity, electrolyte changes, free radical production, inflammation, and delayed apoptosis, which creates an unfavorable microenvironment at the location of injury (2, 3). The duration of secondary injury ranges from minutes to weeks after the initial trauma. Therefore, early clinical intervention to block the mechanisms of secondary spinal cord injury is crucial for effective treatment. The microenvironment is the environment for normal metabolic and functional activities of spinal cord tissue cells, which is mainly composed of neuronal cells, glial cells, immune cells, various cytokines, and factors such as local blood supply, oxygen concentration, pH, and ionic balance. This microenvironment can be categorized into two types: the immune microenvironment, consisting of immune cells, inflammatory factors, and chemokines, and the non-immune microenvironment, consisting of non-immune cells, blood supply, oxygen concentration, ionic balance, and acid-base balance. The immune-inflammatory response persists at the injury site after SCI, involving the microglia, macrophages, neutrophils, lymphocytes, astrocytes and the substances they release, and various inflammatory factors, along with the degradation of the extracellular matrix. These factors significantly contribute to the imbalance of the spinal microenvironment. However, our understanding of the spinal cord microenvironment after SCI remains quite limited (4–6).

The “microenvironmental imbalance” after SCI has been defined as the loss of homeostatic balance in tissue, cellular, and molecular at different times and sites, exacerbating and accelerating the course of SCI (7). It can be subdivided into intra-neuronal and extra-neuronal homeostasis (8). Functional repair of SCI has always been a difficult challenge, the key task to restore neurological function is reshaping and restoring cellular homeostasis. The “microenvironmental imbalance”, particularly the abnormally activated immune microenvironment, poses a crucial obstacle to spinal cord injury repair. Implanting biological materials or functional scaffolds into the damaged site as a therapeutic strategy to restore the SCI microenvironment has shown promising prospects for improving motor function in animal models and severe SCI patients (9–15). However, constructing the physiological microenvironment for spinal cord regeneration using biological materials and molecular drugs (such as 3D printing technology or hydrogels) remains challenging. Issues such as poor cell activity and insufficient interaction between cells and materials leading to incomplete cell differentiation still cannot be adequately resolved (13, 16). Recent studies have proposed a system for studying the interaction between human motor neurons and the microenvironment, providing a new paradigm for studying human neural aging (17). The combination of single-cell and spatial multi-omics (ST) technologies may help in developing precise strategies to repair the spinal cord microenvironment and optimize comprehensive SCI treatment.

## 2 Single-cell technique reveals heterogeneity of the spinal cord injury microenvironment

**Figure 1:**
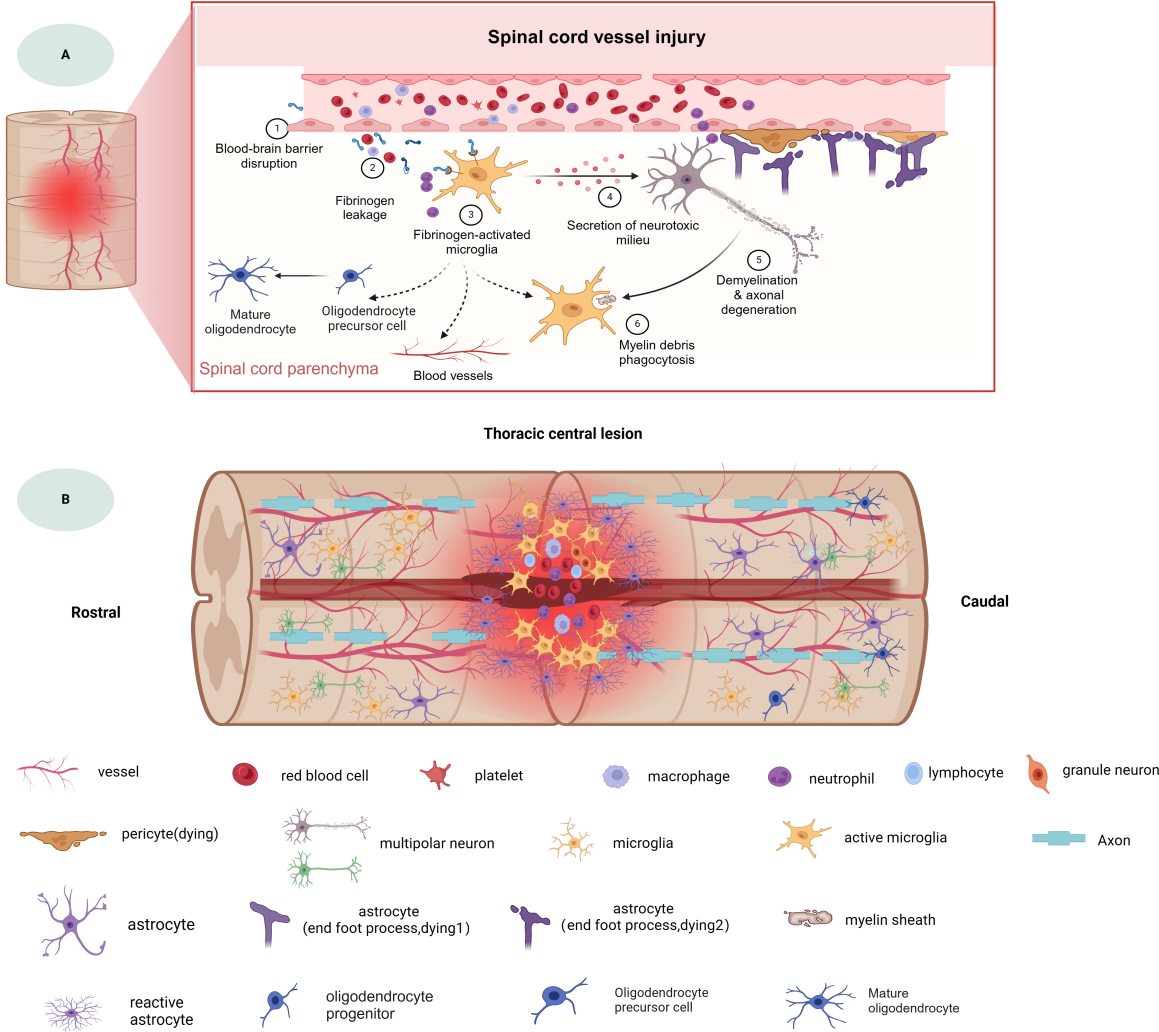
The spinal cord injury microenvironment and vessels landscape revealed by spatial multi-omics. **(A)** Schematic diagram of spinal cord vessel injury, **(B)** Schematic diagram of thoracic central injury.

As mentioned above, the microenvironment after tSCI is complex (**Figure 1**). Different cellular and non-cellular components change over time. The evolution of neuropathology after human spinal cord injury has been reported to be in the chronological order of pro- and anti-inflammatory cellular mediators (18). Different pathophysiological processes are active in the spinal cord parenchyma during the acute phase of the response to SCI (19). Lesion size is widely recognized as the most reliable prognostic predictor after central nervous system (CNS) injury, but similarly sized lesions may produce varying degrees of functional impairment and subsequent recovery. The neuroanatomical functional paradox seems to explain the different clinical outcomes of patients with similar degrees of SCI at the tissue level (20). However, at the cellular level, we are still unclear about the dynamic cellular responses and heterogeneity of the different regions beneath the lesion after SCI.

Differences between individual cells can also have far-reaching functional implications. Cellular heterogeneity is reflected in multiple dimensions, including genomics, transcriptome, epigenetics, proteomics, and metabolomics. Traditional Bulk-RNA sequencing provides only the average gene expression of all but different cell types in spinal cord tissues, which does not allow for the confirmation of heterogeneity in individual cells at the DNA level and the precise revelation of specific variations in each cell type (21–23). In contrast, single-cell RNA sequencing (scRNA-seq) completely overcomes such obstacles, enabling high-resolution and unbiased analysis of neurons, glial cells, T cells, myeloid cells, monocytes, and macrophages after SCI (24–26). Therefore, this technique has been widely used to investigate the heterogeneity of different cells in the development and disease processes of rodent and human spinal cords, providing new insights into different cell types in healthy and diseased tissues (9, 27–32).

Through comprehensive analysis of single-cell and spatial multi-omics data, Li and his colleagues established a cellular map of human spinal cord development and revealed how specific genes regulate cells and their spatial localization, and that human spinal cord development is characterized by unique events (31). Studies have shown that immune remodeling after SCI significantly affects survival and differentiation after stem cell transplantation and the prognosis of SCI (33, 34), as the stem cell-loaded hydrogel scaffold mimics the properties of the natural ECM, it establishes an anti-inflammatory immune microenvironment by reducing inflammation, which promotes neuronal differentiation of stem cells. After SCI, the immune response is not confined to the spinal cord parenchyma, the immune cells of the spinal cord meninges also change. Furthermore, inflammation and immune response exhibit gender and age dependency at the levels of cellular recruitment and gene expression (35, 36). Moreover, there is molecular diversity within the autonomic and skeletal motor neurons as well as the cholinergic neuron types in the spinal cord (21, 37), suggesting that motor neuron senescence and neuroinflammation with overactivation of microglia are important factors in spinal cord aging (17). The ependymal cell is a layer of cuboidal or columnar epithelial cells lining the luminal surfaces of the ventricles and the central canal of the spinal cord, with cerebrospinal fluid secretion, supportive and regenerative roles, and is thought to be the likely endogenous neural stem cell of the adult spinal cord (38, 39).

To better understand the spatial correlation with prognosis, researchers used immunohistochemistry (IHC), immunofluorescence (IF), and laser capture microdissection-based IHC/IF, as well as ancillary tools to select areas of injury to analyze the expression patterns of protein and RNA transcripts (40). In addition, flow cytometry combined with scRNA-seq allows for high-purity classification of specific cell types. In the following sections, we will review recent developments in spatial multi-omics, research applications for exploring histopathologic processes after SCI, and current challenges.

### 2.1 Spatial multi-omics technology

Single-cell RNA sequencing (scRNA-seq) has advanced understanding of the pathophysiology of spinal cord injury, offering insights into new cell types/subtypes, neuroimmunology, biological states, and their activation, enabling the investigation of individual cell behavior, mechanisms, and their relationship with the organism to become a reality. The advantage of sequencing the transcriptome from the single-cell level over traditional bulk ribonucleic acid (RNA) sequencing methods that analyze a population of cells as a unit is its ability to present the molecular profile of RNA within a single cell at an unprecedented resolution while addressing the inability to analyze cellular heterogeneity that is not achievable with sequencing of tissue samples (41). Transcriptome sequencing at the single-cell level currently consists of scRNA-seq, snRNA-seq (41) and spatial transcriptomics (ST) (42). However, both scRNA-seq and snRNA-seq require the dissociation of tissue samples, which implies the loss of interrelationships between cells. Spatial transcriptomics using *in situ* hybridization (ISH) and *in situ* sequencing (ISS) allows for the spatial information of cells to be explored (40). Meanwhile, it preserves the spatial information of the sample by bypassing tissue dissociation (42, 43).

**Figure 2:**
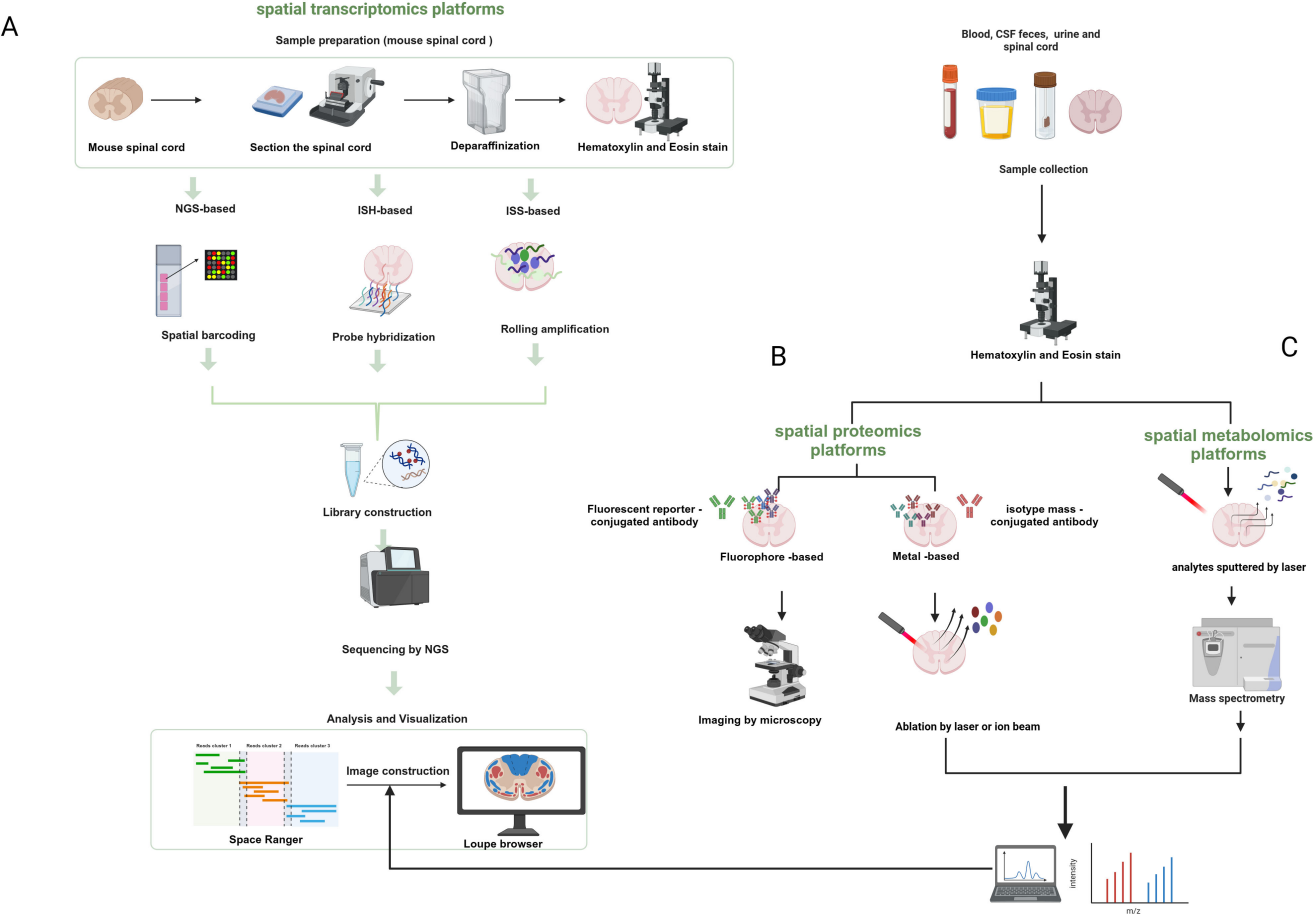
Schematic diagram of the workflow of spatial transcriptomics, proteomics, and metabolic profiling. **(A)** Next-generation sequencing-based platforms and spatial transcriptomics platforms based on in-situ hybridization and in-situ sequencing. **(B)** Spatial proteomics platforms that use a fluorescent reporter molecule or a metal-conjugated antibody to identify target proteins. **(C)** Spatial metabolomics that involves the sputtering of dots or pixels from tissues by lasers that are then ionized and detected for metabolites. Created with BioRender.com.

As mentioned earlier, spatial transcriptomics can provide spatial location information but not at the single-cell level of resolution, whereas single-cell transcriptomics can achieve single-cell resolution but lacks spatial location information. Therefore, combining spatial omics with single-cell omics can improve the resolution and accuracy as well as the spatiotemporal characteristics of the study of tissues and organs of organisms. Moreover, multi-omics characterization of fresh-frozen (FF) or formaldehyde-fixed paraffin-embedded (FFPE) tissue sections can be achieved by combining these spatial single-omics approaches. Spatial multi-omics technology, listed by Nature as one of the seven technologies to focus on in 2022 (44), permits the study of different molecular analytes at up to subcellular resolution in a natural tissue context. More studies are moving away from single “omics” to the use of “multi-omics” to characterize disease progression, which can effectively compensate for the shortcomings of single “omics” and may reveal more biological information than the sum of the individual analyses (45). Arguably, the development and innovation of spatial multi-omics technologies is based on a range of established spatial single-omics approaches (**Figure 2**).

### 2.2 Spatial transcriptomics

Spatial transcriptomics refers to the visualization and quantification of the transcriptome at spatial resolution in a single tissue section (46, 47), allowing gene expression to be assessed in thousands of cells in the context of the structural organization of tissues, which provides a good platform for probing information about the relationship between spatial tissue organization and dysregulated molecular networks in the proximity of pathogenic markers. Previous spatial transcriptomics techniques use either spatial indexing or imaging-based approaches to measure and quantify *in situ* mRNA molecules (48). The former is based on polyA hybridization and is capable of probing the entire transcriptome in an unbiased manner, but the mRNA in the sample needs to remain intact. Imaging-based methods, on the other hand, achieve multiplexing by using fluorescent labeling of *in situ* mRNA molecules and high-resolution fluorescence microscopy to detect and distinguish individual mRNA transcripts, which are subjected to a cycle of labeling, imaging, and signal removal. Currently, the main strategies for spatial transcriptomics are based on next-generation sequencing (NGS) and fluorescence *in situ* hybridization (FISH) (47, 49, 50). Sequencing-based methods involve taking 10-100 μm tissue regions to capture mRNA, and deconvolution of single-cell libraries to infer single-cell contributions (51). Using NGS technology, transcripts are encoded with positional information prior to sequencing. For example, using the 10× Genomics Visium platform (46), microarrays containing spatially barcoded oligomers (dTs, deoxythymidine sequence) were used to capture mRNAs from chip-covered tissues, which were then subjected to a sequencing process, resulting in unbiased spatial transcriptome data. In areas of high cell density, each point may include the transcriptome of a homogeneous or heterogeneous mixture of up to 15-20 cells (28). However, the small number of transcripts captured from tissues covered by microarray chips containing spacer barcode oligomers, and the clustering of transcripts from multiple adjacent cells for downstream analysis, effective spatial resolution cannot reach the single-cell level (52, 53).

### 2.3 Spatial proteomics

Proteins are the molecules that best reflect cellular function and can provide a more important dimension of information for disease characterization. The center of multi-omics is currently shifting from structural genomics to phenomics, including proteomics and metabolomics. Proteomics can reflect information about the complete set of proteins made by a cell or organism, providing information to further understand health and disease development. However, characterizing proteins remains challenging because of the greater chemical diversity in protein composition compared to nucleic acids and because proteins cannot be amplified (54). In the past, protein measurements have relied on antibody-based methods. Previous proteomic analyses required the isolation of cells from suspensions or tissues, which resulted in the loss of spatial contextual information on cell-to-cell interactions between normal and diseased tissues. Until the advent of spatial transcriptomics in 2020 (55), this problem could be solved by FISH or sequencing-based methods (56). Mund et al. (57) proposed that lost spatial information can be obtained by the combination of ultrasensitive mass spectrometry proteomics techniques with multiplex imaging and spatial transcriptomics, and summarized four spatial proteomics-based methods, including multiplex imaging, FISH-based spatial transcriptomics, sequencing-based spatial transcriptomics, deep visual proteomics (DVP), and spatial MS-based proteomics, which each have their advantages. In particular, spatial MS-based proteomics is suitable for the preservation and analysis of proteins from FFPE samples. Overall, single-cell analysis with relatively low throughput remains very challenging (57). Antibody-based multiplex imaging methods include antibody labeling (e.g., metal tags, fluorophores, DNA oligonucleotide barcodes, or enzymes) and detection modalities (e.g., MS, spectroscopic, fluorescent, or colorimetric methods) (58), which can be used for the detection of proteins and mRNAs within the same tissue (59).

### 2.4 Spatial metabolomics

The metabolome, as the active part of the cell, provides a better response to the state of the cell at the time of disease, and single-cell metabolomics is considered by Nature as one of the seven technologies to watch in 2023 (54). Metabolomics is a research method that characterizes lipids, carbohydrates, and other small-molecule metabolites in cells or tissues. Many metabolomics laboratories use dissociated cells that are captured in capillaries and analyzed individually using mass spectrometry. Mass spectrometry (MS) is a robust technique for multiplexed analysis of proteins, natural products, and metabolic derivatives (60, 61), playing a central role in multi-omics strategies (62). Since classical mass spectrometry methods do not provide spatial information, in contrast, mass spectrometry imaging (MSI) is a powerful tool that is an alternative to antibody-based methods. In contrast to traditional optical microscopy techniques, MSI allows not only the spatial characterization of proteins or peptides, but also the simultaneous and sensitive label-free detection, quantification, and characterization of hundreds of other small biomolecules, such as lipids, metabolites, or glycans in tissues and cells, in tissue slices (63). Bender et al. (64) have developed a new technique to maintain the spatial structural integrity of the spinal cord, using H&E staining and MSI can show differences in chemical composition. To date, matrix-assisted laser desorption/ionization (MALDI) remains the most popular ionization technique (65). MALDI-MSI enables spatial metabolome characterization of tissue sections at (near) single-cell resolution, capturing various information on the spatial distribution of cellular metabolites in different parts of the sample (54, 66).

Spatial multi-omics technologies are now widely used to study the transcriptome, proteome, and metabolome of the immune microenvironment of spinal cord injury (67), it has enabled new insights into the interactions between intracellular and inter-cellular molecular mechanisms that control development, physiology, and pathogenesis (68–70). We summarize the application of this technique in clinical trials and animal experiments, and next, we review the progress of research on this technique to reveal the microenvironment of spinal cord injury (**Table 1**).

**Table 1:** Overview of researches on multi-omics study of spinal cords.

Reference	Method	Target	Species(Sex/Age)	Model	Tissue source	cell cluster	Tissue preparation
**(Li et al., 2023a)** (31)	10x Visium	Sequencing	Human and mice (embryonic fetal)	fetal spinal cords	human prenatal and mouse fetal spinal cords	Intermediate neuronal progenitors excitatory neurons inhibitory neurons cholinergic neurons astrocytes ependymal cells OPCs oligodendrocytes	FF
**(Gong et al., 2023)** (28)	10x Visium	Sequencing	C57BL/6J mice( female/8-wk)	T10 hemi-section SCI	Injured spinal cord at 3dpi,7dpi,14dpi and 28dpi	microglia macrophages astrocytes oligodendrocytes fibroblasts endothelial cells	FF
**(Hakim et al., 2021)** (26)	10x Chromium	Sequencing	C57BL/6J mice (female/10-15wk ) and human (ND)	T10 contusion SCI	Uninjured and injured spinal cord at 0.5 h, 2 h, 6 h, 24 h, 36 h, 3 d, 7 d, 21d and 90d	CD45 cell Microglia macrophage monocyte macrophages dendritic cell B cell	FFPE
**(Matson et al., 2022)** (71)	10x Chromium	Sequencing	C57BL/6 mice ( female / 12–30wk)	T9 contusion SCI	Uninjured and injured lumbar spinal cord and 1wpi,3wpi,6pwi	OPCs MOL NFOL	FF
**(Wang et al., 2022)** (72)	10x Chromium	Sequencing	C57/BL6 mice(three-month-old )	T10 contusion SCI	Uninjured and injured spinal cord at 0dpi,3dpi,14dpi	microglia macrophage monocytes monocyte-derived cells neutrophils mast cells monocyte-derived cells dendritic cells Natural Killer cells NK T cells T cells B cells endotheliocyte monocyte-derived fibroblast	FF
**(Floriddia et al., 2020)** (73)	MERFISH FISSEQ	Sequencing	Mice(ND)	T10 dorsal funiculi transection SCI and T9 contusion SCI	Injured spinal cord at 14 dpi,3mpi,5mpi	Oligodendrocytes MOLs OPCs MOL2 MOL5 MOL6	FF
**(Wu et al., 2023)** (74)	10x Chromium	Sequencing	Mice (ND)	T10 contusion	Uninjured and injured spinal cord at 1dpi ,3dpi,7dpi	oligodendrocytes OPCs OPC1 OPC2 OPC4 COPs MOLs NFOL1-2 MFOL1-2 MOL1-6	FF
**(Ikeda-Yorifuji et al., 2022)** (41)	10x Chromium	Sequencing	C57BL/6 J mice (female /7wk)/ and neonate mice (female / P1)	T10 hemi-section SCI	Injured spinal cord at 7dpi	neuron astrocyte oligodendrocytes OPCs microglia endothelial cell pericyte contacting neuron	FF
**(Hou et al., 2022)** (25)	10x Chromium	Sequencing	C57/BL6 mice (two months old)	T10 heme-section SCI	Uninjured and injured spinal cord at 0.5dpi,1dpi,3dpi,7dpi,14dpi, 60dpi,90dpi	microglia, macrophages, granulocytes endothelial cells epithelial cells erythrocytes monocytes astrocytes oligodendrocytes B cells T cells	FF
**(Hara et al., 2017)** (75)	Illumina HiSeq	Sequencing	C57/BL6 mice (female/6-8wk)	T10 contusion SCI	Uninjured and injured spinal cord at 7dpi,14dpi	astrocytes reactive astrogliosis	FF
**(Milich et al., 2021)** (76)	10x Chromium	Sequencing	C57BL/6J mice (female /8-10 wk)	T8 contusion SCI	Uninjured and injured spinal cord at 1dpi,3dpi,7dpi	neutrophils monocytes macrophages microglia dendritic cells pericytes ependymal cells endothelial cells oligodendrocytes OPCs	FF
**(Fan et al., 2023)** (9)	Illumina NovaSeq 10x Chromium	Sequencing	rhesus monkeys (female/ 4-7 years)	T9 transection SCI	Injured spinal cord at 0 dpi,7 dpi,14 dpi,30 dpi and 6 months post injury	Neurons microglia macrophages OPCs astrocytes ependymal cells fibroblasts pericytes endothelial cells Schwann cells T cells	FF
**(Brennan et al., 2022)** (77)	10x Chromium	Sequencing	C57BL/6 J mice (female /8–10 wk)	T9 contusion SCI	Uninjured and injured spinal cord at 7dpi,28dpi	Microglia MDMs endothelial cells monocytes astrocytes T cells ependymal cells B cells intermediate progenitors neutrophils erythroid cells oligodendrocyte lineage pericytes leptomeningeal cells	FF
**(Li et al., 2022)** (27)	10x Chromium	Sequencing	C57BL/6 mice (male and female /8wk)	T9 crush SCI	Injured spinal cord at 15 min, 1 d, 3 d, 7d, 14 d, 28 d, and 42 d after injury	stromal cell neutrophils lymphocytes ependymal cells oligodendrocytes pericytes erythrocytes OPCs endothelial cells neurons astrocytes microglia	FFPE
**(Wang et al., 2023)** (78)	10x Chromium	Sequencing	salamanders (male and female /10 months old)	SCI induced by marrow destruction method	Injured and uninjured spinal cord at 0dpi,4dpi,10dpi	neuron	FF
**(Zhang et al., 2023)** (79)	10x Chromium	Sequencing	C57BL/6 mice (female)	T9 clamp SCI	Injured and uninjured spinal cord at 3dpi,7 dpi	Astrocytes B cells endothelial cells ependymal cells granulocytes microglia macrophages monocytes NK cells oligodendrocytes	FF
**(Salvador et al., 2023)** (36)	10x Chromium	Sequencing	C57BL/6J (female/2-4 months of age or 18-24 months of age)	T9 contusion SCI	Injured spinal cord at 0 dpi,3dpi,7 dpi, and 14dpi	microglia monocytes neutrophils DCs T cells NK cells B cells Stromal cell macrophages	FFPE
**(Martins et al., 2023)** (80)	10x Chromium	Sequencing	C57BL/6J mice (female/8–9 wk)	T9 contusion SCI	Injured and uninjured spinal cord at 0dpi,3 and 7 dpi	endothelial cells, pericytes and perivascular macrophages	FF
**(Yao et al., 2022)** (81)	10x Chromium	Sequencing	Sprague Dawley rats (female)	T10 contusion SCI	Injured and uninjured spinal cord at 0dpi,3 dpi,7 dpi	microglia endothelial cell macrophage neutrophil pericyte fibroblast vSMC astrocyte erythrocyte oligodendrocyte precursor cell B cell monocyte oligodendrocyte T cell	FF
**(Skinnider et al., 2021)** (19)	Mass Spectrometry(MS)	Protein	Yucatan miniature pigs (female /150 to 200 days) and human (ND)	T10 contusion SCI	Spinal cord at the first 5 dpi from patients and 15 min prior to injury, and at 12 h, 24 h, 48 h, 72 h, 120 h and 12 weeks post injury	CSF and serum samples	FF
**(Li et al., 2023b)** (82)	MIBI(Multiplexed Ion Beam Imaging)	Protein	Wistar rats (female /8wk)	T10 contusion SCI	Injured spinal cord at 7dpi	Proteins and phosphorylated proteins	FF
**(Devaux et al., 2016)** (83)	MIBI	Protein	Rats (ND)	T7-T11balloon compression SCI	Injured at 3dpi,7dpi, 10dpi and uninjured spinal cord	Protein	FF
**(Yao et al., 2021)** (84)	MS	Protein	Sprague-Dawley rats (male)	C5 hemi-contusion SCI	Injured uninjured spinal cord at 3 dpi,7 dpi ,14dpi	Protein	FF
**(Pang et al., 2022)** (85)	MS	Metabolite	Wistar rats (female /8wk)	T10 contusion SCI	Injury and uninjured spinal cord at 4 h, 24 h and 48 h post SCI	arachidonic acid	Dried sample
**(Zeng et al., 2022)** (86)	MS	Metabolite	Sprague-Dawley rats (male)	T10 hemi-section SCI	Uninjured and injured at 3dpi	purine metabolism	FF
**(Yang et al., 2022)** (87)	MS	Metabolite	Sprague–Dawley rats (female)	T10 contusion SCI	Injured and uninjured spinal cord at 72h	plasma and CSF, spinal cord	FF
**(Chen et al., 2013)** (88)	MS	Metabolite	Long-Evans rats (female)	T10 contusion SCI	uninjured and injured spinal cord at 24 dpi	proteins involved in ubiquitination, endocytosis and exocytosis, energy metabolism, inflammatory response, oxidative stress, cytoskeletal disruption, and vascular damage	FF
**(Calvo et al., 2024)** (89)	MS	Metabolite	C57BL/6 mice (male/14wk)	LPC-induced demyelination SCI	Injured spinal cord at 14dpi	Lipidome	FFPE
**(Graham et al., 2023)** (90)	MS	Metabolite	C57BL/6 mice (male/4-month-old)	T10 transection SCI	Uninjured 7dpi blood serum	Serum lipid	FF
**(Wu et al., 2016)** (91)	MS	Metabolite	Human (ND)	SCI patient(AIS A/B/C)	24h,48h,72h after SCI	Cerebrospinal Fluid and Serum	FF

Wk, week; P1, postnatal day 1; ND, not determined; dpi, days post injury; wpi, weeks post injury; mpi, months post-injury; FF, Fresh frozen; FFPE, Formalin-fixed, paraffin-embedded; OPCs, Oligodendrocyte precursor cells; COPs, Committed oligodendrocyte progenitor cells; MOL, Myelinating and mature oligodendrocytes; NFOL, Newly formed oligodendrocytes; vSMC, Vascular smooth muscle cells; LPC, Lipopolysaccharide.

## 3 Neuronal dynamics after SCI

Recent studies have identified motor neurons as the most sensitive cell type to senescence in the spinal cord, as evidenced by a significant increase in cellular senescence-related markers as well as regression of neuronal function (17). Regenerative genes were partially activated in neurons near the lesion during the acute phase, whereas neurons in distal tissues showed a weaker response in the early phase (9). Sathyamurthy et al. (92) reveal differences in the spinal cord between the ventral and dorsal neuron populations. The greatest changes in cell type after SCI were in neurons, astrocytes, and microglia, with a decrease in the relative abundance of neurons and astrocytes and an increase in the relative abundance of microglia. Similarly, oxidative damage to neurons in the core of the lesion declines in the months following SCI and remains present at the margin of injury until 1.5 years after SCI (93). Although the most dramatic changes were seen in the non-neuronal population after SCI, neurons showed a more delayed response (71). Excitatory and inhibitory interneuron proportions changed in the proximal and distal regions of the lesion, with an increase in the fraction of excitatory interneurons at the proximal lesion site, a decrease in inhibitory neuron proportions, and no significant changes in proportions at the distal lesion site. Unlike neurons at the thoracic lesion site, neurons at the lumbar lesion site distal to the injury were largely preserved (71). Furthermore, spatial and temporal and spatial changes in oxidase-2 (COX-2) and 5-lipoxygenase (5-LOX) after spinal cord injury occur mainly in neurons in the central, cephalic, and caudal parts of the spinal cord (85). In addition, during SCI the spinal cord neurons show autoimmune characteristics. Studies have shown that interneurons and motor neurons transiently express immunoglobulins (IgGs) at 3 days post-SCI. Differences in IgGs expression in different neuronal populations may be due to their different responses to the extent and severity of injury. Moreover, injection of anti-CD20 did not attenuate the expression of IgGs detected at 3 days post-injury(dpi) and reveal any improvement in motor function compared with SCI without anti-CD20 treatment at 0, 1, 3, 7, 14, 21days until 28 days after SCI (83).

## 4 Astrocytes dynamics after SCI

Astrocytes are organic components of the CNS and are distributed throughout the CNS, performing many functions essential for normal neuronal development, synapse formation and remodeling, neural circuit function, and action potential propagation, interacting with microglia to maintain homeostasis in the CNS (94–97). They provide energy and neurotransmitters to neurons to maintain homeostasis and act as a physical barrier between the synaptic connections of neighboring neurons (98). After SCI, astrocytes function in an activated manner to remove necrotic tissues, form protective barriers, maintain microenvironmental homeostasis, interact with immune cells, and form glial scarring astrocytes. SCI-induced astrocytes are heterogeneous. For example, neuroinflammation and ischemic responses induce astrocytes to differentiate into inflammatory astrocyte 1 (A1) and neuroprotective astrocyte 2 (A2) types (99, 100). A1 astrocytes upregulate the expression of complement cascade genes, which disrupt synapses and are detrimental to the nervous system. In contrast, A2 astrocytes upregulate the expression of various neurotrophic factors, and promoting the conversion of A1 astrocytes to A2 astrocytes can be neuroprotective (101, 102). Reactive astrocytes are astrocytes that undergo morphological, molecular, and functional remodeling as a result of CNS injury, disease, or infection, eventually becoming reactive astrocytes that promote scarring. However, the process of reactive astrocyte generation impedes axonal regeneration and nerve repair (103).

Hou et al. (25) constructed transcriptomic profiles of mouse astrocytes, including Atp1b2+, S100a4+, Gpr84+, C3+/G0s2+, GFAP+/Tm4sf1+, and Gss+/Cryab+ astrocytes, from uninjured spinal cord tissues and injured tissues adjacent to the center of injury at different time points, and combined with immunofluorescence staining depicted the spatial distribution of different astrocyte populations at the injury site (71). In contrast, GFAP + reactive astrocytes increase rapidly after SCI, peaking around day 1, then declining but remaining above non-injurious levels up to 42 days after SCI (27). Reactive astrocytes will eventually transform into scar-forming astrocytes, impairing axonal regeneration and functional recovery. Blocking the interaction between reactive astrocytes and type I collagen can prevent the formation of astrocytic scars, promoting axonal regeneration and functional improvement (75). Although most glial scar cells are GFAP-positive, their proliferation is not necessary for the development of glial scarring. In a spinal cord regeneration in salamanders, despite the presence of GFAP-positive cell proliferation, no neuroglial scar formation was observed, and the site of injury was eventually populated by mature neurons and GFAP-positive neuroglia in the spinal cord membrane, which facilitated the restoration of motor function in salamanders (78).

## 5 Microglia dynamics of after SCI

Microglia are macrophages that help maintain CNS homeostasis through continuous interactions with neuronal and non-neuronal cells (104). In the healthy primitive spinal cord, microglia make up the largest proportion of CD45+ immune cells (36). A major challenge after spinal cord injury is to differentiate between microglia and monocyte-derived macrophages (MDM), and transcriptomic studies have revealed microglia-selective genes to a certain extent, with the core of the lesion after SCI containing predominantly blood-derived monocytes/macrophages, which further exhibit both pro- and anti-inflammatory phenotypes, whereas microglia within the rim locally proliferate and display predominantly pro-inflammatory features (93). Zhang et al. determined that B2m and Itgb5 are predominantly located in microglia while Vav1 is predominantly located in macrophages by combined scRNA-seq and bulk RNA seq techniques. Decitabine is a hypomethylating agent. It hypomethylates DNA by inhibiting DNA methyltransferase (105). They found that low-dose decitabine may promote spinal cord regeneration by modulating macrophage/microglia polarization status (79), targeting of Hif1α promotes microglia regeneration and functional recovery after SCI (72). Microglia are intrinsic myeloid cells of the CNS, which can be categorized into homeostatic microglia (bMicroglia), inflammation-activated microglia (aMicroglia), dividing microglia (dMicroglia), interferon-activated microglia (iMicroglia) based on GO analysis (26), and monocyte-activated microglia (maMicroglia) (76). More than 90% of microglia in the undamaged spinal cord are in homeostasis and are the main population of myeloid cells (76). It has been found that microglia are the most responsive cell type to perturbation (71), injury leads to large-scale sustained activation of bmicroglia into a disease-associated state characterized by lipid metabolism and phagocytosis (DAM), and bmicroglia exhibit changes in gene expression after injury (9, 26). There were significant differences in the number and morphology of microglia after SCI. At 1 dpi, the fraction of bmicroglia declined to 20% (76), was replaced by microglia subtypes with different transcriptional profiles, and microglia accumulated in lumbar spinal cord tissue 8 cm from the injury. Quantification of microglia showed that the highest densities of Iba-1(a microglia-specific marker) positive cells were observed in gray matter areas of the caudal region at 1 and 3 days after SCI, but there was no difference in Iba-1-positive activated microglia in rostral (R) and caudal (C) spinal cord tissues away from the site of injury. Differences in microglia densities between the R and C segments were only observed at 3 and 7 days after SCI. Subsequently, the density of microglia in either segment decreased at 7 dpi, but remained higher than the basal microglia density observed in controls (83). During the 3-7dpi, microglia in the gray matter of the spinal cord caudal to the injury site is notably more active than those in the white matter, expressing various levels and forms of cell surface antigens (106, 107), whereas the activation of microglia decreased at 10 dpi (83). Until 30 days after injury, the number of microglia was still increasing. CDH13 is necessary for cell–cell adhesion and tissue integrity in adult organisms, and functions as membrane receptors mediating signals received from the extracellular space. Almost all microglia did not express CDH13, even in distal lumbar spine tissue 6 months after SCI (9). After SCI, microglia undergo a rapid transition to down-regulate the bMicroglia gene and overexpress GPNMB and CSTD (9), ultimately ceasing in a chronic DAM-like state. Hakim et al. explored changes in immune cell populations over time using scRNA seq to characterize the microglial cell responses at 0.5 h, 2 h, 6 h, 24 h, 36 h, 3 d, 7 d, 21 d, and 90 d after SCI. During the early acute phase, bMicroglia rapidly transformed into aMicroglia after SCI (0.5 h), and then completely transformed into a distinct population of dMicroglia by 2 h after SCI. This transformation ceases between 6 and 36 h, and its state is determined by the expression of Mt2 and Msr1, forming monocyte-activated microglia (maMicroglia) (26). Overall, microglia undergo rapid and profound changes after SCI, eventually arresting in a chronic DAM-like state. Studies have found striking transcriptional similarities between DAM in SCI and DAM in neurodegeneration, demyelination, and development, and microglia depletion experiments have shown that DAM in SCI originates from permanent transcriptional reprogramming of bMicroglia and that DAM also enhances the recovery of motor function after SCI. DAM is usually present 3 days after SCI, and from 3dpi to 21 dpi in increasing numbers and persists during the chronic phase of SCI (26).

Interestingly, in the acute phase, DAM expresses more genes involved in lipid transport in the spinal tissue-retained area (SA) activated by astrocytes. In contrast, DAM away from the region of injury significantly increases the expression of phagocytosis-associated genes, and DAM persists in phagocytosis up to the chronic phase. However, microglial cells in this region do not respond as rapidly as the region of injury (8). Activated microglia A cells (similar to DAM cells) have been reported to remain present in the white matter of the damaged spinal cord at 6 wpi (week post-injury) and even in the lumbar spine tissue distal to the injury, which also suggests that they may play a sustained role at chronic time points (71). Single-cell transcriptomics has also shown that microglia are important for neural repair in SCI in mice (77). Microglia are not only involved in the immune repair response and inflammatory process after injury, but also in the process of neovascularization. A recent study found that microglia could promote endogenous angiogenesis by regulating endothelial cell subpopulations through the SPP1 and IGF signaling pathways after SCI (81), which provides a new target for exploring SCI intervention. Optimal repair after spinal cord injury (SCI) in mice requires key ligand-receptor interactions between microglia, astrocytes, and the MDM, and microglia are required for optimal functional recovery and restoration of lesion homeostasis after SCI. What’s more, activated microglia also occur during aging and the ability of microglia to transition to other subtypes after SCI decreases with age (36).

## 6 Oligodendrocyte dynamics after SCI

In spinal cord tissues, myelin is produced by oligodendrocytes (OLs), and OLs and oligodendrocyte progenitor cells (OPCs) are critical for maintaining myelin morphology and axonal regeneration. Indeed, OLs and oligodendrocyte lineage cells make up the highest proportion of cells in the lumbar spinal cord (108). OPCs originate from distinct ventral and dorsal structural domains within the ventricular germinal zone of the embryonic CNS, and ventral and dorsal spinal cord OPCs differ in their ability to differentiate into mature oligodendrocytes. The enhanced proliferation, recruitment, and differentiation of dorsal OPCs contribute proportionally more to myelin re-formation than in the ventral side. However, the ability of dorsally derived OPCs to differentiate into mature oligodendrocytes decreased with age (up to 13 months). This suggests that the responsiveness of OPCs to demyelination, their contribution to myelin re-formation, and their susceptibility to age-related functional decline are dependent on their developmental starting point in the developing neural tube (109). It was also found that sex-determining region Y (SRY) box 9 (Sox9)-positive cells at homeostasis can differentiate into oligodendrocytes up to two weeks after SCI, and are also involved in the formation of glial scarring (110). Changes in oligodendrocytes (OLs) after SCI are closely related to the microenvironment of the spinal cord, the production of excitotoxic substances in the acute phase leads to the dramatic loss of OLs, while the loss of OLs in the chronic phase is due to the presence of oxidative stress (111). Research indicates that at different time points, microglia in the spinal cord and brain of mice exhibit similar transcriptional features (112, 113). Monocyte transcriptomics on isolated nuclei reveals resident cycling cells contribute significantly to the oligodendrocyte lineage (114). This suggests that OLs and their precursor cells throughout the CNS have different morphologies and are closely associated with functional differences and neural repair in different regions.

The majority of OPCs differentiate into oligodendrocyte precursors (OPCs) and mature oligodendrocytes (MOLs), and a minority of OPCs differentiate into another branch and terminate as MOLs (74). Oligodendrocyte lineage included oligodendrocyte precursor cells (OPCs), cellular progenitors of oriented oligodendrocytes (COPs), newly formed oligodendrocytes (NFOLs), and myelin-forming and maturing oligodendrocytes. The proportion of oligodendrocyte lineage cells did not change significantly after injury, except for a significant increase in COPs at 1wpi compared with those in the uninjured spinal cord in mice (71). Marques et al. (108) identified oligodendrocytes from different regions of the mouse juvenile/adult CNS. They found that the ratio of OPC2 to OPC4 increased significantly at 1 dpi but decreased progressively at 3 dpi and 7 dpi during the acute injury phase of SCI, whereas the fraction of OPC1 and differentiation-induced OPCs showed the opposite trend. It was also found that newly formed oligodendrocytes are responsive to complex motor learning, and different MOL populations have spatial preferences in the CNS. In mice, MOL2 and MOL5/6 showed differential susceptibility in the chronic phase. Unlike the brain, MOL2 is enriched in the spinal cord. MOL5/6 is enriched at the site of spinal cord lesions with age, MOL6 is preferentially present in the white matter and sensory tracts of the spinal cord in mice (71). Additionally, OPCs are also involved in the formation of glial scars. Gong et al. (28) assessed the cellular heterogeneity among oligodendrocytes in the scar and identified three oligodendrocyte subtypes in mice. Subtype1 is mainly distributed in the outer layer of macrophages in the scar core at 3 dpi and diminished rapidly. By contrast, subtype 2 cells were mainly distributed in the white matter of the scar. Subtype3 cells were significantly enriched for pre-synapse and significantly increased at 7 dpi. They also identified some markers for activated oligodendrocytes, including the expression of Mbp, Mog, Mag, and Cldn11. Therefore, the development of single-cell sequencing technology has enabled researchers to obtain highly accurate cellular transcriptional information, facilitating detailed studies of cellular subpopulations and temporal-spatial changes, which can help to understand the mechanisms involved in the injury and repair process.

## 7 Peripheral immune cell dynamics after SCI

Common peripheral immune cells include neutrophils, monocyte-derived macrophages, and lymphocytes. Neither neutrophils nor lymphocytes are normally present in the uninjured spinal cord; but appear in the spinal cord at a specific time after injury. Neutrophils are the first peripheral cells to enter the site of injury after SCI and reach a peak at 24 h after injury (83, 115). Normal spinal cord tissue neutrophils at the rostral and caudal to the injury site showed a large increase in neutrophils, which peaked at 3 pi and then declined over time. In addition, it was found that the morphology of neutrophils was different in the control and SCI groups, with normal neutrophils being elongated and bipolar, and new branches appearing in the cells after SCI (83). At 14 dpi, mature neutrophils are the predominant neutrophil subtype, whereas there is an increase in immature and semi-mature neutrophils, mainly associated with proliferation, angiogenesis and regeneration (115). Early in the injury MDM usually enters the injured spinal cord from the injured spinal cord blood-brain barrier within 2-3 days, peaking with T cells at 7dpi and 9 dpi, and persisting until 180 dpi (116, 117).

By studying monocytes and macrophages after SCI in mice, researchers have found that monocytes and macrophages infiltrate the spinal cord parenchyma and participate in the regulation of inflammation along with microglia, both of which are polarized to either a pro-inflammatory M1 or anti-inflammatory M2 phenotype. However, they shift to the M2 phenotype, exhibiting anti-inflammatory effects during the recovery phase (118). M2 macrophages in mice are consistently located at the periphery of the injury site at 3 dpi, becoming rare at 14 and 28 dpi. The expression of their marker, Arg1, peaks at 3 dpi and then declines rapidly (119). Peripheral macrophages are divided into two major groups, homeostatic macrophages (hMφ) and injury-associated macrophages (IaMφ1, IaMφ2). IaMφ1 and IaMφ2 infiltrate mainly at 3 dpi. IaMφ1 is associated with elevated oxygen levels, respiratory chain, and autophagy, and IaMΦ2 is associated with wound healing and regeneration. Peripheral macrophages control the dynamics of pro-inflammatory cytokines Il-1β and Tnf-α, which promote functional spinal cord regeneration in zebrafish and are required for spinal cord repair (120). During this period, monocytes of local bone marrow origin infiltrate from the dura mater into the spinal cord and mediate the immune response of the central nervous system (121).

In a healthy spinal cord containing a small number of resident B cells, B cells are reduced at 3 dpi after SCI but return to normal levels at 14 dpi (72). Previous studies have reported that antibodies produced by B cells hinder the functional recovery of SCI (122). Although the transcriptional profiles of B and T cells remain essentially unchanged over time, lymphocyte phenotypes alter dramatically. In SCI mice, T cells are the main lymphocytes, Wang et al. (72) divided T cells into αβ T cells and γδ T cells. Among them, CD4+ and CD8+ subsets constitute the bulk of αβ T cells and are the main component of T-mediated immune responses (123, 124). After SCI, the proportion of these cell types changed, and young survivors of quadriplegia have reduced CD4:CD8 ratios (125). During the immune response after SCI, most CD4 T cells are biased towards T helper type 1 (Th1) and T helper type 17 (Th17) cells. αβ T cells appeared at 3dpi, and the αβ T cells appeared at different time points were completely different, producing different cytokines respectively. In contrast, γδ T cells were observed at 14 dpi and were almost always activated Th17 cells. Activated T cells (ICOS T cells or CD39 T cells) and exhausted T cells (CTLA4 T cells) increased at 14 dpi (72). Regulation of the balance among different CD4+ T cell subsets is crucial for repairing the injured spinal cord through the induction of protective immunity in nerve regeneration (126). These results suggest that lymphocytes mainly infiltrated the lesion core restricted by the glial scar.

## 8 Scar dynamics after SCI

The glial scar is known to be a structure that contains a variety of cellular and non-cellular components that inhibit or promote regeneration, as well as being important for limiting and controlling further expansion of tissue damage (**Table 2**). Although different types of cells are proven to be involved in scarring, the lack of a genealogical tracking system for all the corresponding cells makes it difficult to determine which cells play a key role, a problem that can be well addressed by ST technology (127, 128). As mentioned previously, activated astrocytes surround the injured area, while non-neuronal cellular components, including localized and infiltrating immune cells and fibroblasts that produce extracellular matrix, accumulate in the core of the scar and constitute the fibrotic or matrix component of the scar (129). Zhang et al. (128) have described in detail the microglia, astrocytes, and astrocytes that form after scar formation following SCI, spatial distribution and dynamics of fibroblasts and macrophages. It has been shown that environmental changes in the injured spinal cord prevent astrocyte scar formation and promote axonal regeneration (130). The traditional view is that macrophages at the scar site are derived from the blood supply (119), however, recent studies have found that monocytes after SCI may be derived from the adjacent cranial and spinal bone marrow (121). Gong et al. (28) suggested that scarring may go through the following four phases based on the proportion of scarred cells: a macrophage infiltration phase, a scar-resident cells proliferation and differentiation phase, a scar emergence phase and a scar quiescence phase. The revelation of the spatial and temporal dynamics of scar formation can provide a reliable reference for future interventions in scar formation.

**Table 2:** Cellular and non-cellular components of glial scar.

Components	Cell	Type	Molecular/signal	Role
Cellular components	Astrocytes	Reactive astrocyte	STAT-3 signaling pathway and LZK	Mediated proliferation of astrocytes
			Upregulate expression of Nes, Ctnnb1, Axin2, Plaur, Mmp2, Mmp13, CCL2 and Csf2	Contribute to the recruitment of other cell types, primarily immune cells and ensnaring fibroblasts
		A1-like astrocytes	Upregulate Cdh2, Sox9, Xylt1, Chst11, Csgalnact1, Acan, Pcan, Slit2 and secrete TNF-α, IL-1, IL-6, FGF and NGF	Induce neuroinflammation
		A2-like astrocytes	Neurotrophic factors	Protective phenotype
	Microglia	M1 microglia(primary)	Interferon-γ, LPS-TLR4 and secrete IGF-1, TNF-α, IL-1β, IL-6 and IL-12	Induce the switch of microglial cells to the M1 type and initiate glial scar formation
		M2 microglia	IL-4, IL-13, IL-10 and TLRs and secrete IL-10 and IL-13	Induce microglial cells to switch from M1 to M2 type
	Blood-derived monocytes/macrophages	Ly6C^hi^ monocytes subtypes	CCL2, CCL5, CXCL8	phagocytic and pro-inflammatory type
		Ly6C^lo^ monocytes subtypes	CCL2, CCL5, CXCL8	Anti-inflammatory type
		M1-like macrophages	None	Prevent cell proliferation
		M2-like macrophages	TGF-β	Promote cell proliferation
	OPCs	None	Express NG2 (gene name cspg4) and PDGFRα	Inhibits axon growth, differentiate into astrocytes and oligodendrocytes
	Ependymal cells	Differentiate into astrocytes or oligodendrocytes	None	Differentiating primarily into astrocytes
		Differentiate into neurons	BDNF/TrkB-MEK/ERK signaling pathway,sirtuin 2 (SIRT2)	Facilitate the differentiation of ependymal cells towards neurons
	fibroblast	None	None	Produce stromal ECM molecules and fortify the glial scar structure
Non-cellular components	Intermediate silk proteins	GFAP, nestin, vimentin protein	Astrocyte	Stabilize the glial scar
	ECM	CSPGs, fibronectin, laminin, collagen, and proteoglycans	Fibroblasts and astrocytes	

IFN-γ, interferon-gamma; TNF- α, tumor necrosis factor-alpha; IL, interleukin; OPCs, oligodendrocyte progenitor cells; GFAP, glial fibrillary acidic protein; FGF, fibroblast growth factor; NGF, nerve growth factor; CSPG, Chondroitin Sulfate Proteoglycan; PDGFRα, platelet-derived growth factor receptor-α; LZK, leucine zipper-bearing kinase; ECM, Extracellular matrix.

## 9 Analysis of vascular heterogeneity after SCI

A hallmark of secondary SCI is disruption of vascular continuity and a reduction in the number of vessels at the site of injury, leading to decreased oxygen delivery and loss of mitochondrial homeostasis and further cellular damage. Subsequent infiltration of peripheral inflammatory cells and release of reactive oxygen species further exacerbate the injury (70, 131). This leads to a loss of autoregulation in the injured segments of the spinal cord and a significant reduction in microcirculation in the gray and white matter. Studies have shown that the loss of microcirculation extends over a considerable distance proximal and distal to the injury site (3). However, the formation of endogenous neovascularization after SCI is limited. In the normal spinal cord, blood vessels are predominantly found in the gray matter. After SCI the density of blood vessels in the gray matter decreases and spreads rostral and caudal to the site of injury. Smith et al. (110) investigated the temporal and spatial changes of the microvessels in rats at 2dpi, 5dpi, 15 dpi and 45 dpi and showed a significant decrease in microvessels density in the ventral and dorsal gray matter in the center of the injury and the three spinal cord segments surrounding the spinal cord at 2dpi. Neovascularization was observed at and around the injury site at 7 dpi and persisted until 45 dpi (110). In addition, BSCB was abnormally leaky after SCI, with differential expression of different genes by endothelial and pericytes, mainly at the center of the lesion and in the caudal region of the injury, in a spatially dependent manner (80). Notably, in normal spinal cord microglia have little contact with blood vessels, but after SCI BSCB was abnormally leaky, endothelial and pericytes express different genes in a spatially dependent manner (80) microglia activate and migrate to the injured area after SCI, and immune cells such as macrophages regulate endothelial subpopulations to promote endogenous angiogenesis via the SPP1 and IGF signaling pathways, which provides new clues for exploring angiogenic interventions in SCI (81). In addition, there are strong interactions between endothelial cells and fibroblasts. In addition to the canonical vascular signaling pathway VEGFA-FLT1, fibroblast ligands such as FN1 and collagen are also involved in endothelial cell interactions (28). Overall, vascular transcriptomic analyses from acute to chronic time points provide insight into microvascular remodeling after SCI, which facilitates the identification of key vascular therapeutic targets for improving future functional repair after SCI.

## 10 Proteomics after SCI

Mass spectrometry and methodological techniques with advances in bioinformatics have greatly improved the accuracy of the detection of small compounds such as proteins and various types of metabolites (including lipids) from biological tissues. These proteomics- or metabolomics-based approaches allow the identification of molecular signatures associated with changes in physiological systems, which in turn allows the identification of mechanisms targeted by interventions. While proteomics is suitable for high-throughput analysis of protein expression differences across multiple samples or treatment conditions, spatial proteomics techniques can reveal the spatial expression patterns of proteins after SCI, and understand how proteins are involved in complex biological processes in the different environments of cells in the injured tissues may help to unravel potential biomarkers and underlying mechanisms of SCI (84).

Proteomic analysis of cerebrospinal fluid and blood from pigs and humans showed significant changes in CSF protein abundance after SCI, with C-reactive protein being one of the most strongly altered proteins, which is consistent with previous findings (88). However, a less pronounced time course was seen in serum, suggesting that the serum proteome reflects a delayed systemic response to SCI. Qu et al. (132) used proteomic techniques to reveal the association between T10 SCI-induced neurogenic bladder sphincter overactivity and synaptic vesicle glycoprotein (Sv2A), which is involved in the release of neurotransmitters from synaptic vesicles, and the involvement of the α-adrenergic receptor and G protein-coupled receptor internalization, the level of arrestinβ2 inhibitory protein, and calmodulin and calmodulin-binding proteins involved in calcium-sensitive signaling pathways were strongly correlated, but no spatial information was revealed. Spatial proteomics reveals that various chemokine and cytokine levels are also significantly altered in acute SCI and that such changes correlate with the site of injury (83). In the SCI rat model, levels of ubiquitination, the ras-related protein Rab-3A (Rab3A) associated with cytosolization and cytokinesis, proteins associated with energy metabolism (mitochondrial matrix enzymes, glutamate dehydrogenase (GDH), and fructose bisphosphate aldolase C), and C-reactive proteins showed a significant increase in levels at 24 hours post-injury, whereas levels of cytoskeletal proteins were significantly reduced at 24 hours post-injury (88). Protein changes one week after SCI mainly involved the GABA ergic synaptic pathway, the ErbB signaling pathway, tight junctions, adhere junctions and those related to phosphorylation processes, these proteins have a strong correlation (82).

## 11 Metabolomics after SCI

As the end products of cellular activities, metabolites are closer to the phenotype, and subtle changes in genes and proteins are amplified in metabolites. Currently, metabolomics is increasingly used as an indicator for early diagnosis, prognostic assessment and monitoring of treatment response in cancer and metabolic diseases (133–135). Similarly, there is increasing interest in the role of metabolomics in SCI, particularly in studies related to specific biomarkers for assessing the severity and prognosis of SCI (87, 91). Studies have shown that metabolites in cerebrospinal fluid and serum after SCI can reflect injury severity to some extent (19, 87, 91), and can be used to monitor injury progression and treatment response in animal studies. In rats choline phosphate, pyridoxine and guanidino acetic acid can be used as potential metabolite biomarkers for SCI severity assessment, whereas in humans there are six metabolites in the CSF after SCI whose levels are affected by injury severity, including citrulline, glycerol, lactate, N-acetyl putrescine, N1, N12 diacetyl spermine, and N-methyl-D-aspartic acid, whereas in the serum only 5-hydroxy lysine correlated with injury severity (91). In addition, glyoxylate and dicarboxylic acid metabolic pathways in spinal cord tissue were most relevant to the SCI response (87). Metabolic analysis of cerebrospinal fluid more accurately reflects metabolic changes in spinal cord tissue than plasma (87, 88, 91). Dysregulation of arginine-proline metabolism after SCI is directly related to SCI injury in humans (91). Peng et al. (136) created a “characteristic metabolome” based on metabolomics profiling by 1H-NMR that was sufficient to differentiate between rats with severe SCI and healthy control rats. They found that metabolites could affect neurobehavioral recovery. Studies have shown significant changes in serum lipid abundance 7 days after SCI in mice but minimal changes in the metabolome (90). Pang et al. (85) determined by arachidonic acid metabolomics that the acute phase in SCI rats is characterized by an upregulation in the expression of cyclic COX-2 and 5-LOX and is accompanied by an increase in the expression of prostaglandin E2 (PGE2) and leukotriene B4 (LTB4) levels increased, and that PGE2/COX2 and LTB4/5-LOX may provide novel strategies for SCI repair. In addition, lipid changes in SCI mice tend to occur around the site of injury rather than in the center of the injury, suggesting the presence of an active demyelination process (89). Metabolomics has also revealed that dietary ω-3 fatty acid-derived metabolites are associated with chronic pain responses after SCI in rats (137). These studies suggest that illustrating the effects of metabolic dynamics on the CNS after SCI may be useful for repair.

## 12 Conclusion and prospect

Studies using spatial multi-omics tools have revealed the complexity of time and have shown that in addition to cellular composition, the relative position and interactions of cell types in the cellular microenvironment strongly influence the development of tissue damage (69). In conclusion, the development of spatial multi-omics has led to a qualitative leap in precision medicine and personalized treatment, especially for oncology patients (138). Moreover, ST has the potential to discover new cellular biomarkers. For example, Glmp and Nfe2l2 are DAM-specific transcription factors in SCI (26). Sun et al. (17) suggested that CHIT1 could be used as a humoral marker to measure age in the primate spinal cord, whilst human cerebrospinal fluid and serum protein biomarkers is related to injury severity or prognosis, and protein abundance at 48 hours usually has the greatest prognostic value (19). Not only that, ST may also reveal the mechanism of promoting SCI repair based on biomaterials such as scaffolds and hydrogels (95). These studies help us to establish biochemical markers that can predict neurological outcomes in animal models and human patients, allowing the results of preclinical studies to inform the diagnosis and prognosis of human SCI. However, we must also consider the limitations of animal studies. Human spinal cord development is unique compared to rodents (Li et al., 2023a), and there are obvious differences between the two in spatiotemporal gene expression. In addition, there are significant temporal differences between mice and rats.

**Figure 3:**
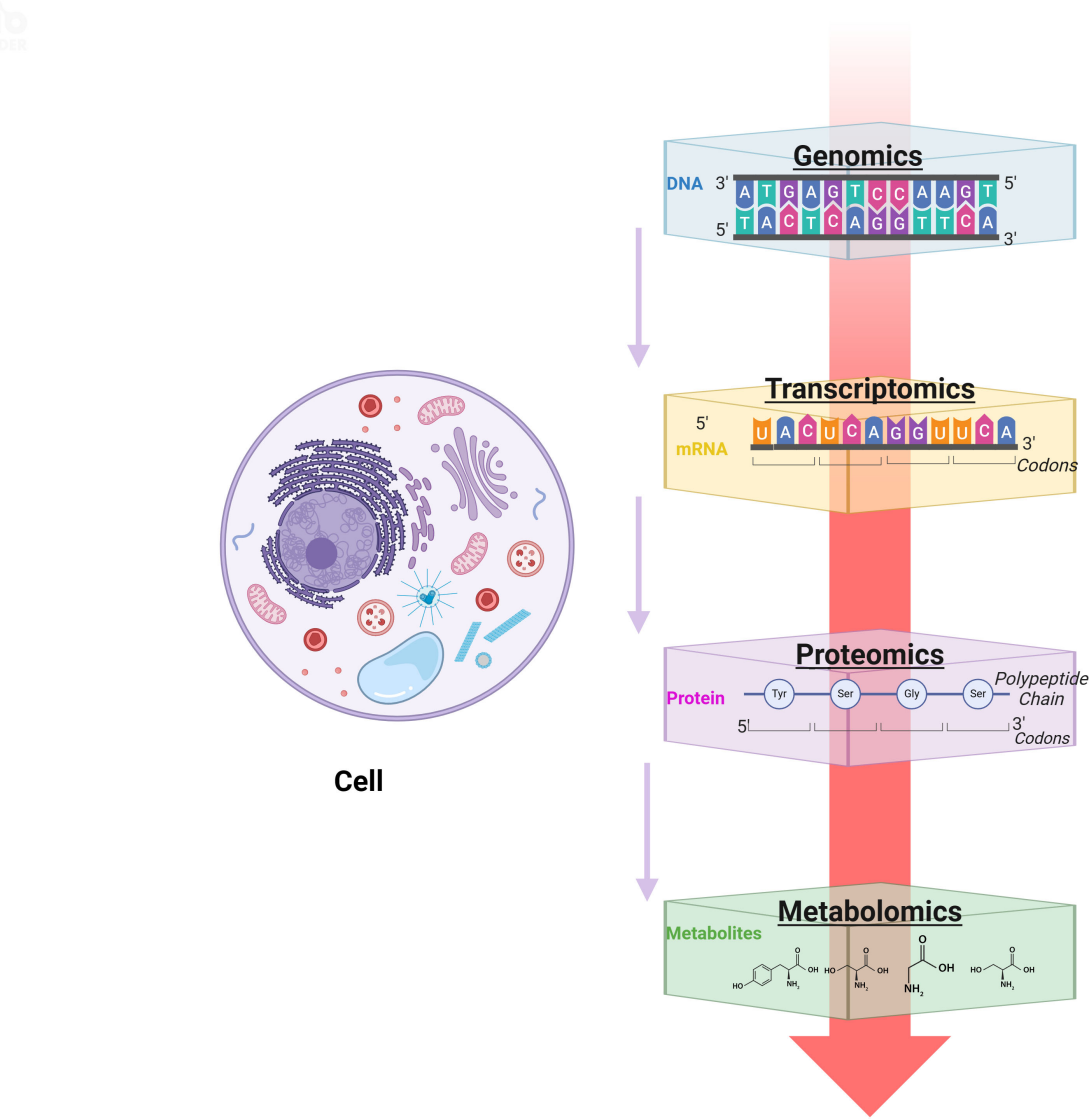
Single-cell technology research idea map. Created with BioRender.com.

However, most of the current studies are still based on a single omics. In the future, we should conduct more comprehensive “multi-omics” studies on the spatiotemporal development of the SCI mechanism and self-repair (**Figure 3**), including single-cell genomics plus transcriptomics, single-cell epigenomics plus transcriptomics, single-cell genomics plus low-throughput analysis of another analyte, spatial (epigenomics) genomics plus transcriptomics, spatial transcriptomics combines proteomics, and other combinations of analytical strategies (45, 67), to explore a series of molecular changes involved in the development of SCI in the spinal cord at the levels of genomes, transcriptomes, proteomes, and metabolomes. These approaches can deepen our understanding of the underlying mechanisms of SCI progression, which is crucial for exploring potential therapeutic approaches and finding new therapeutic targets.
